# Histochemical and Semi-Quantitative Evidence of Dimoxystrobin-Induced Liver Extracellular Matrix Remodelling in Adult Zebrafish

**DOI:** 10.3390/jox16040148

**Published:** 2026-08-11

**Authors:** Ilaria Olivito, Valentina Basile, Antonio Paolo Maria Graziani, Naouel Gharbi, Rachele Macirella

**Affiliations:** 1 Department of Biology, Ecology and Earth Science (DiBEST), University of Calabria, Via P. Bucci 4/B, 87036 Rende, Italy; ilaria.olivito@unical.it (I.O.); valentina.basile@unical.it (V.B.); antoniopaolomaria.graziani@unical.it (A.P.M.G.); 2 Fish Biology and Aquaculture Group, Ocean and Environment Department, NORCE Norwegian Research Center, 5006 Bergen, Norway; nagh@norceresearch.no

**Keywords:** hepatotoxicity, collagen deposition, glycosaminoglycans, hepatobiliary alterations, pesticide toxicity

## Abstract

Strobilurin fungicides are among the most widely used agrochemicals worldwide. Dimoxystrobin has been shown to induce hepatic injury in adult zebrafish; however, whether this damage also involves early extracellular matrix remodelling and hepatobiliary remodelling remains unknown. Here, we investigated early remodelling of the extracellular matrix, focusing on initial collagen deposition and glycosaminoglycan redistribution after a short-term exposure (96 h) to two environmentally relevant concentrations of dimoxystrobin (6.56 and 13.13 µg/L) on the liver of *Danio rerio* using an integrated histological, histochemical, and semi-quantitative approach. The results showed a progressive dose-dependent alteration of hepatic architecture, accompanied by a significant increase in bile pigment-like deposits and collagen deposition. Exposure also induced a significant increase in the deposition of acidic glycosaminoglycans and a severe depletion of glycogen-dependent Periodic Acid–Schiff positivity components, accompanied by an increase in amylase-resistant Periodic Acid–Schiff reactive components. Overall, these findings indicate that dimoxystrobin-induced hepatotoxicity extends beyond parenchymal degeneration to encompass coordinated stromal and hepatobiliary remodelling. The present study also indicates that the periductal microenvironment may represent a sensitive target of dimoxystrobin toxicity and supports the use of histochemical and semi-quantitative analyses as sensitive tools for detecting sublethal pesticide-induced early extracellular matrix remodelling in fish liver.

## 1. Introduction

Fungicides are a major class of pesticides, widely used in agriculture to protect crops against fungal infections that can reduce yield and compromise agricultural production [1,2,3]. Demand for fungicides has grown alongside population growth; nevertheless, the widespread application of these compounds may pose risks to animal, human, and environmental health [4,5]. Strobilurins are an important class of fungicides widely used in agriculture, accounting for 20–25% of the global market [6,7]. These compounds belong to the quinone outside inhibitor (QoI) class and exert their antifungal activity by inhibiting the mitochondrial cytochrome bc1 complex, thereby reducing ATP production [8,9,10]; however, since mitochondrial respiration is evolutionarily conserved across taxa, they may also affect non-target organisms [11].

Dimoxystrobin, a second-generation strobilurin fungicide, is characterised by notable environmental persistence, with prolonged degradation times in soil and aquatic matrices [12,13]. Due to its widespread detection and potential threat to aquatic ecosystems, dimoxystrobin was included in the 3rd Watch List (WL) under the Water Framework Directive in 2020 and maintained in the 2022 update (Commission Implementing Decisions EU 2020/1161 and EU 2022/1307). Concerns over the ecotoxicological safety of dimoxystrobin ultimately led to the withdrawal of its approval in the European Union in 2023, owing to risks to non-target organisms and the potential for groundwater contamination by its metabolites [13]. Nevertheless, dimoxystrobin continues to be commercialised in several non-EU countries such as Brazil. Moreover, withdrawal of regulatory approval does not necessarily result in the disappearance of a substance from the environment, because pesticide residues and their transformation products may remain in soil, sediment, groundwater, and surface waters, raising substantial concerns about their risks to aquatic biota [14,15]. Dimoxystrobin has adverse effects on honeybee larvae [13] and has been classified as highly toxic to aquatic organisms, with long-term effects [16]; however, available regulatory and ecotoxicological data have mainly focused on acute toxicity tests on a few aquatic invertebrates and fish [17], leaving sublethal tissue-level mechanisms under-investigated in fish species.

The zebrafish (*Danio rerio*) is a prominent vertebrate model in ecotoxicology [18,19,20] and is one of the few fish species with a fully sequenced and publicly available genome [21]. It has many critical physiological pathways that are highly conserved in higher vertebrates, and over 82% of genes associated with human diseases have functional orthologs in zebrafish [22,23,24]. It has been recognised as a powerful tool for evaluating the health risks posed by environmental pollutants to both aquatic biota and mammals [19].

Recent data using this model have shown that dimoxystrobin induces developmental alterations, compromises swimming performance, and modulates genes involved in the mitochondrial respiratory chain in both embryos and larvae [25]. In addition, in adult zebrafish, exposure to this fungicide leads to systemic disturbances, including gill injury and hepatic degeneration associated with oxidative stress and metabolic imbalance [26,27]. In fish, the liver is the primary hub for xenobiotic biotransformation and lipid homeostasis, making it a critical target for environmental toxicants [28,29,30]. Our previous findings on dimoxystrobin hepatotoxicity in adult zebrafish provided important insights into parenchymal injury, showing severe hepatic degeneration, metabolic storage alterations and oxidative stress induction [27]. However, whether this injury also involves early remodelling of the stromal extracellular matrix and hepatobiliary remodelling remains unknown.

Liver injury is a multicellular process that extends beyond hepatocyte death, involving dynamic alterations in the extracellular matrix (ECM) and the hepatobiliary compartment [31,32]. Following hepatic injury, localised cell death and the subsequent inflammatory response are accompanied by ECM damage [32,33,34]. If the damage persists, liver regeneration can fail, leading to progressive structural remodelling and the accumulation of fibrous connective tissue [35]. While this tissue-level response is well documented in mammalian models [36,37,38,39], the early phases of ECM remodelling, such as initial collagen deposition and glycosaminoglycan redistribution, remain poorly understood in fish [40].

To address this gap, the present study investigated, for the first time, early remodelling of the extracellular matrix, focusing on initial collagen deposition and glycosaminoglycan redistribution along with hepatobiliary alterations in the liver of *Danio rerio* after 96 h of exposure to 6.56 and 13.13 µg/L of dimoxystrobin, through a histological, histochemical, and semi-quantitative approach. The selected concentrations are environmentally relevant since they fall within the range of dimoxystrobin concentrations in surface waters worldwide (0.10 ng/L–16.42 μg/L) [41,42].

Specifically, Hematoxylin and Eosin (H&E) staining was performed to establish a baseline histopathological profile, evaluating general parenchymal architecture, circulatory disturbances, and inflammatory foci. Periodic Acid–Schiff (PAS) staining, performed with and without amylase digestion, was used to evaluate hepatic glycogen depletion and to distinguish glycogen-dependent staining from amylase-resistant PAS-positive structures. Alcian Blue pH 2.5 staining was used to detect acidic glycosaminoglycans, whereas Azan Trichrome staining was applied to evaluate early collagen deposition. Finally, Fouchet–Van Gieson staining was used to detect bile pigment-like deposits as a possible indicator of altered hepatobiliary clearance.

Overall, the results presented herein provide new evidence on the hepatotoxic effects of dimoxystrobin on fish, shifting the focus from isolated hepatocellular injury to early stromal–parenchymal and hepatobiliary disorganisation, and propose histochemical and semi-quantitative methods that may improve the assessment of pollutant-induced hepatotoxicity in aquatic vertebrates.

## 2. Materials and Methods

### 2.1. Fish Husbandry and Maintenance

Adult wild-type AB zebrafish aged 6–8 months, of both sexes, were housed in the zebrafish facility of the Department of Biological Science at the University of Bergen. Water parameters were strictly monitored and maintained at constant levels: temperature of 26–28 °C and a photoperiod of 14 h light and 10 h dark. The animals were fed twice daily with live brine shrimp (*Artemia salina*). The experiment was approved by the Norwegian Food Safety Authority (permit number: FOTS ID 29916, date of approval: 20 January 2023), and it was performed as previously described in Macirella et al. [27].

### 2.2. Exposure Solution

Dimoxystrobin (analytical grade, purity ≥98.0%; Sigma-Aldrich Chemical Co., Gillingham, UK) was utilised to prepare a stock solution by dissolving 1000 µg of the fungicide in 100 µL of acetone, which was subsequently diluted in 1000 mL of facility system water. The stock solution was further diluted with facility system water to obtain the two sublethal, environmentally relevant concentrations selected for this study: 6.56 µg/L (low concentration) and 13.13 µg/L (high concentration). The concentrations of dimoxystrobin selected in the present study correspond to 40% and 80% of the Predicted Environmental Concentration of this fungicide in freshwater (PECfw = 16.42 μg/L), respectively [41]. The control group received an equivalent volume of vehicle (acetone < 0.001% *v*/*v*). Analytical verification of dimoxystrobin in water samples was performed as previously described by Ahmed and colleagues [25]. Briefly, dimoxystrobin concentrations in the exposure water were verified by UV–visible spectrophotometry using a Varian Cary 50 Scan spectrophotometer. Absorbance was measured at 220 nm using a 1 cm quartz microcuvette. Quantification was performed by external calibration with a dimoxystrobin analytical standard (Sigma-Aldrich Chemical Co., Gillingham, UK; CAS 149961-52-4) prepared in water/acetonitrile (5:2, *v*/*v*). Five standard concentrations ranging from 1.63 to 26.37 mg/L were analysed in triplicate. For each exposure condition, 250 mL of water was evaporated to dryness, and the residue was reconstituted in 600 µL of the same solvent mixture used for the standards. Dimoxystrobin concentrations were calculated from the calibration curve and corrected for the concentration factor. The measured concentrations were 6.58 ± 0.10 µg/L and 13.66 ± 0.33 µg/L for the low- and high-concentration groups, respectively.

### 2.3. Experimental Design and Sampling

An *a priori* power analysis was performed to determine the minimum number of animals required (G*Power 3.1.9.7 software; Franz Faul, Universität Kiel, Germany). A total of 42 fish were randomly assigned to three experimental groups (14 fish per group): control, low-concentration, and high-concentration. Each experimental group initially consisted of seven males and seven females, ensuring equal sex ratios. However, sex was not retained as a sample-level variable for the subsequent histological and histochemical analyses. Animals were housed in 30 L tanks. The exposure (96 h) was conducted under a static regime with daily renewal of the test solutions. During the exposure period, fish were not fed, in accordance with the OECD guidelines [43], and water quality parameters were recorded daily immediately before renewal of the test solutions and maintained at constant levels. Each experimental group included three replicate tanks, which were considered the experimental units. At the end of the exposure, fish were first euthanised by immersion in buffered MS-222 (Sigma-Aldrich Chemical Co., Gillingham, UK—0.20 mg/mL) and then transferred to an ice-water bath to ensure hypothermic shock (0–4 °C).

### 2.4. Tissue Preparation

For histological and histochemical analyses, liver samples from seven animals for each exposed group were fixed in Bouin’s solution for 24 h at 4 °C. After fixation, the tissues were rinsed, dehydrated through a graded ascending series of ethanol, cleared in xylene, and embedded in paraffin wax (melting point 56 °C). Serial sections were cut at a thickness of 5 µm using a rotary microtome (Leica RM2125 RTS; Leica Microsystems, Wetzlar, Germany), mounted on glass slides, and stored for subsequent staining.

### 2.5. Histochemical Staining

All histochemical stains and commercial diagnostic kits were supplied by Bio-Optica (Milan, Italy). The staining procedures were performed according to the manufacturer’s instructions. Prior to all staining, the 5 µm paraffin sections were deparaffinized in xylene and rehydrated through a decreasing ethanol series to distilled water.

For Hematoxylin and Eosin (H&E) staining, rehydrated, deparaffinized sections underwent routine staining in accordance with standard laboratory protocols.

For Alcian Blue pH 2.5 staining (code 04-160802, Bio-Optica, Milan, Italy), rehydrated deparaffinized sections were incubated with 10 drops of Reagent A for 30 min. Slides were then drained without washing, immediately covered with 10 drops of Reagent B, and left for 10 min. After rinsing in distilled water, 10 drops of Reagent C were applied for 5 min. Following a final rinse in distilled water, sections were dehydrated, cleared, and mounted.

For Azan Trichrome staining (code 04-001802, Bio-Optica, Milan, Italy), rehydrated deparaffinized sections were placed in a Coplin jar containing Reagent A and incubated in an oven at 56 °C for 30 min. After cooling at room temperature for 5 min and rinsing with distilled water, the sections were covered with 10 drops of Reagent B for 1 min and then with 10 drops of Reagent for 1 min, without intermediate washing. Slides were then incubated with 10 drops of Reagent D for 30 min, and with 10 drops of Reagent F for an additional 30 min. Finally, sections were quickly rinsed in 95% ethanol, dehydrated in an ascending series of ethanol, cleared with xylene, and mounted.

For Fouchet–Van Gieson staining (code 04-121872, Bio-Optica, Milan, Italy), rehydrated deparaffinized sections were incubated for 5 min with a freshly prepared mixture of 5 drops of Reagent A and 5 drops of Reagent B. After rinsing in distilled water, sections were covered with 10 drops of Reagent C for 7 min. Without washing, the slides were dried with filter paper and air-dried for 5 min. Finally, the sections were left in 100% ethanol for 15 s, cleared in xylene, and mounted.

Periodic acid–Schiff reaction and a predigestion test with α-amylase were performed to detect glycogen (code 04-130803, Bio-Optica, Milan, Italy). The α-amylase predigestion test was performed prior to the PAS reaction to compare with enzyme-untreated sections. Rehydrated, deparaffinized sections were covered with 10 drops of Reagent A (α-amylase) at room temperature for 10 min to enzymatically digest glycogen deposits. After several washes in distilled water, the sections were treated with 10 drops of Reagent B followed by 10 drops of Reagent C for 20 min. After sequential treatment with Reagent D and Reagent E for 2 min each, nuclear counterstaining was performed with Reagent F for 3 min. Sections were finally rinsed in tap water for 5 min, dehydrated, cleared, and mounted. Parallel sections not subjected to amylase treatment were used as a reference to qualitatively assess total glycogen depletion. Image analysis was performed by the same investigator using identical threshold settings for all images.

### 2.6. Semi-Quantitative and Statistical Analyses

Stained sections were examined under an optical light microscope, DM4 B (Leica Microsystems, Wetzlar, Germany), equipped with a high-resolution digital camera, K3C (Leica Microsystems, Wetzlar, Germany). For the semi-quantitative evaluation of histochemical staining, liver sections from seven fish per experimental group, including the control group, were analysed. For each animal, five liver sections were observed and photographed, and non-overlapping microscopic fields were acquired under the same magnification and imaging conditions. Preliminary statistical analysis (Kruskal–Wallis test) confirmed that there were no significant differences among replicate tanks within the same experimental group (*p* > 0.05; Tables S1–S5). Therefore, individual fish from different replicate tanks were considered independent biological observations and pooled for subsequent analyses. Image analysis was performed using ImageJ 1.54n software (NIH, Bethesda, MD, USA). For each histochemical reaction, the positively stained area was quantified using a colour threshold [44]. Results were expressed as the percentage of positively stained area relative to the total tissue area.

For each animal, data obtained from the five analysed sections were used to obtain representative individual values for each histochemical endpoint. Statistical analyses were performed using GraphPad Prism 8.00 software (GraphPad Software, Inc., San Diego, CA, USA). Data normality was assessed using the Shapiro–Wilk test, whereas homogeneity of variances was evaluated using Bartlett’s test. Because the data satisfied the assumption of normality but not that of homoscedasticity, statistical comparisons among groups were performed using Welch’s one-way analysis of variance, followed by Dunnett’s T3 multiple-comparisons test. Differences were considered statistically significant at *p* < 0.05.

## 3. Results

### 3.1. Hematoxylin–Eosin Staining

**Figure 1:**
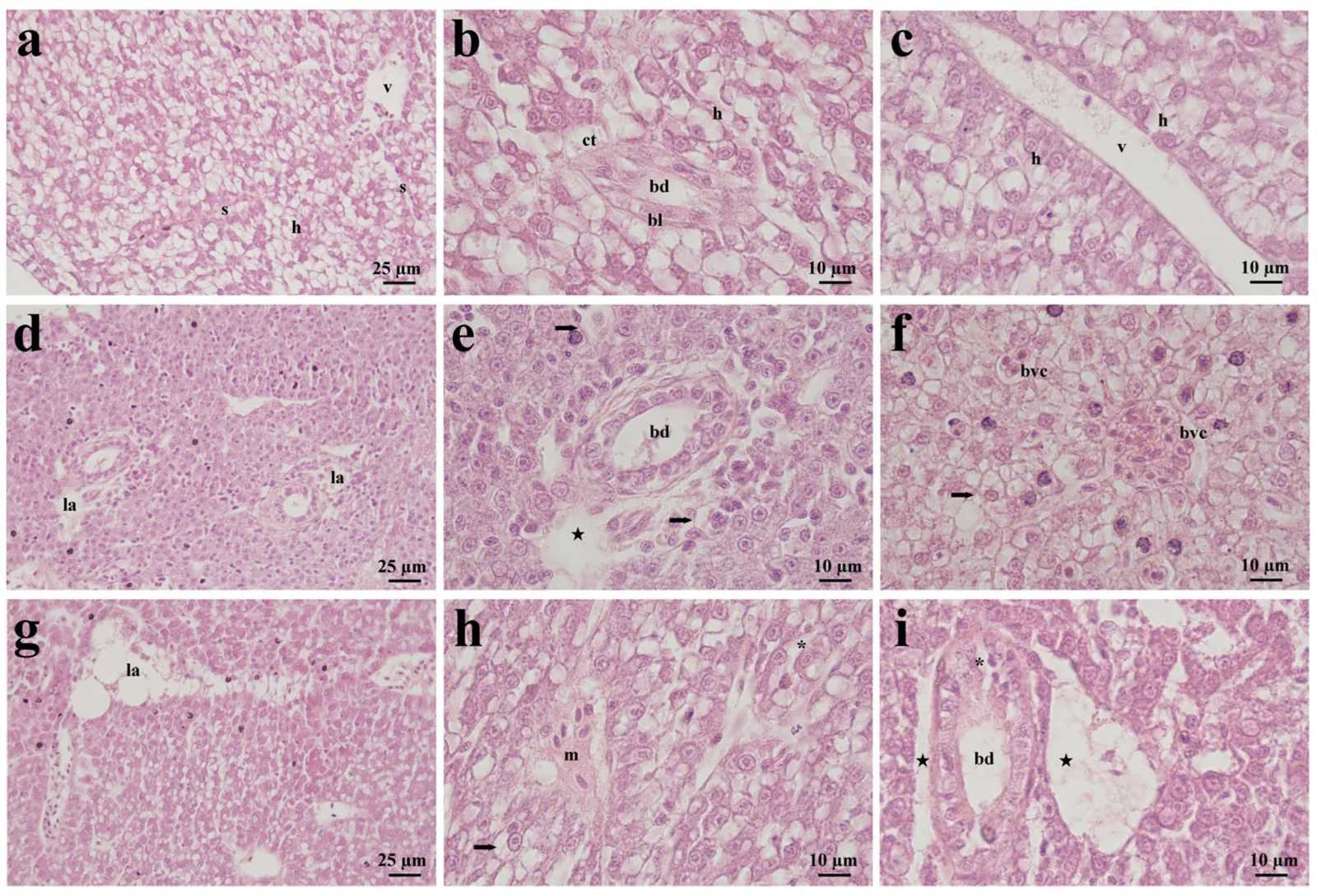
Light micrographs of *Danio rerio* liver (H&E-stained section). (**a**–**c**) General organisation of the homogeneous parenchyma and stromal compartments under basal conditions. (**d**–**f**) After 96 h of exposure to 6.56 µg/L of dimoxystrobin, the parenchyma was heterogeneous, with extensive areas of lysis. Note the swelling of hepatocytes, the bile duct epithelial detachment and the congestion of blood vessels. (**g**–**i**) After 96 h of exposure to 13.13 µg/L of dimoxystrobin, structural alterations were more frequent and severe, with extensive lysed areas that led to the loss of parenchymal structural continuity. Alterations such as bile duct epithelial detachment and degeneration of hepatocytes and cuboidal cells were severe; moreover, mononuclear cell infiltration was also observed. bd = bile duct, s = sinusoid, v = vein, h = hepatocyte, ct = connective tissue, bl = basal lamina, la = lysed area, m = mononuclear cell infiltration, star = bile duct epithelial detachment, bvc = vessel congestion, arrow = swelling of hepatocytes, asterisk = degenerated cell. The original microscopy images are provided in Supplementary Materials, File S1.

In the control group, the liver exhibited a well-preserved structural organisation clearly distinguishable into parenchymal (i.e., hepatocytes and hepatocyte cords) and stromal components (i.e., bile ducts, blood vessels and sinusoids). The parenchyma appeared homogeneous and compact, composed of polygonal hepatocytes arranged in cords with their basal surface oriented toward the sinusoids (Figure 1a–c). Hepatocytes displayed distinct cellular boundaries, eosinophilic cytoplasm, and centrally located round nuclei with evenly distributed chromatin (Figure 1b,c). Within the parenchyma, sinusoids were visible as pale intercellular spaces separating hepatocyte cords (Figure 1a). Bile ducts appeared as circular structures lined by a simple cuboidal epithelium, surrounded by a well-defined basal lamina and a thin connective tissue layer (Figure 1b). Blood vessels were bordered by a continuous endothelium with a few erythrocytes within the lumen (Figure 1c).

Exposure to the low concentration of dimoxystrobin induced structural alterations affecting both parenchymal and stromal compartments. The hepatic parenchyma partially lost its homogeneous appearance, showing evident disorganisation of hepatocyte cords. Wide, pale spaces, also called lysed areas, were observed within the tissue, interrupting the compact arrangement of the liver parenchyma (Figure 1d). Several hepatocytes appeared swollen, with pale cytoplasm (Figure 1e,f). The stromal compartment also displayed marked modifications. The epithelium lining the bile ducts appeared partially detached from the underlying connective tissue, and in some cases, its integrity was compromised, as evidenced by irregular cellular alignment (Figure 1e). Blood vessels frequently appeared congested, with numerous erythrocytes visible within their lumens (Figure 1f).

In fish exposed to the high concentration of dimoxystrobin, alterations observed in the low-concentration group were present with greater severity and frequency. Although the hepatic parenchyma remained identifiable, the tissue appeared markedly heterogeneous. Hepatocyte cords were irregularly arranged, and large areas exhibited loss of structural continuity (Figure 1g). Some hepatocytes showed advanced degenerative features, with pronounced variability in size and shape, enlarged cytoplasm, and reduced eosinophilia (Figure 1h). The stromal compartment was also severely affected. Bile ducts showed pronounced epithelial detachment and irregular cellular distribution, resulting in a thickened appearance; moreover, some cuboidal cells were degenerated (Figure 1i). In this group, mononuclear cell infiltration was also observed (Figure 1h).

### 3.2. Fouchet–Van Gieson Staining

**Figure 2:**
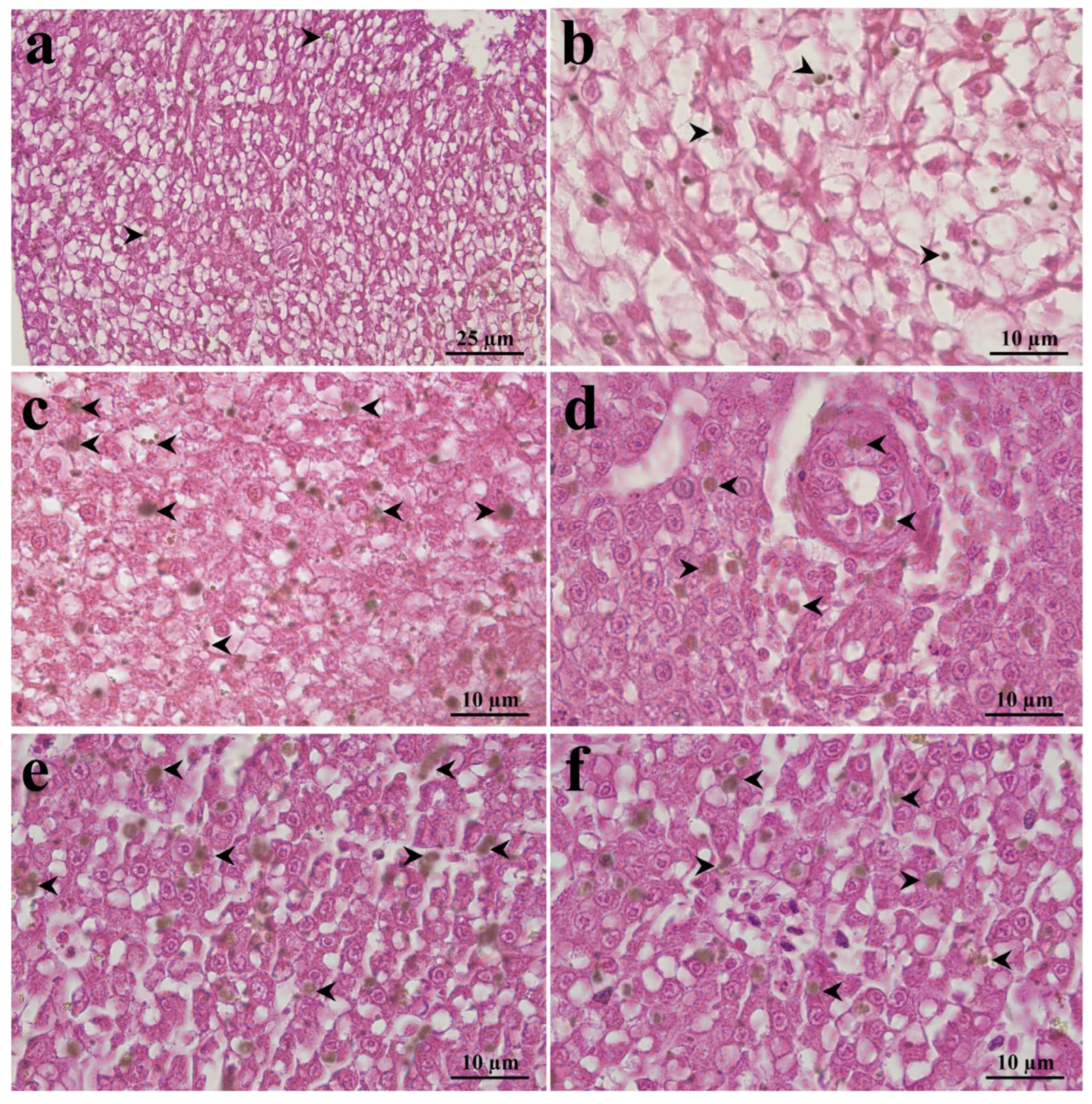
Light micrographs of *Danio rerio* liver (Fouchet–Van Gieson staining). (**a**,**b**) In the control group, slight greenish-brown pigment deposits were visible in the cytoplasm of a few hepatocytes (arrows). (**c**,**d**) After 96 h of exposure to 6.56 µg/L of dimoxystrobin, increased green-brown deposits were observed within hepatocytes and within epithelial cells lining the bile ducts (arrows). (**e**,**f**) After 96 h of exposure to 13.13 µg/L of dimoxystrobin, the staining further increased in the cytoplasm of hepatocytes and was noted in the sinusoids and blood vessels (arrows). The original microscopy images are provided in Supplementary Materials, File S1.

In the control group, the hepatic parenchyma showed slight bile pigment-like deposits within the cytoplasm of a few hepatocytes. The deposits were evenly distributed, without evidence of clustering; the stromal compartment appeared negative to the reaction (Figure 2a,b).

Exposure to the low concentration of dimoxystrobin resulted in a significant increase (*p* < 0.001) in bile pigment-like deposition in both parenchymal and stromal compartments (Figure 2c,d; Table 1). Compared with control, bile pigment-like deposits became more abundant and were distributed throughout a large number of hepatocytes (Figure 2c). Bile pigment-like deposits were occasionally detected within epithelial cells lining the bile ducts (Figure 2d). Blood vessels and sinusoids remained negative to the reaction.

In fish exposed to the high concentration of dimoxystrobin, bile pigment-like deposits significantly increased (*p* < 0.001) and were more extensive and widespread compared to both the control and the low-concentration groups (Figure 2e,f; Table 1). Numerous hepatocytes exhibited intense cytoplasmic accumulation (Figure 2e). In this group, scattered bile pigment-like deposits were particularly evident in the sinusoids and areas adjacent to blood vessels (Figure 2f).

### 3.3. Azan Trichrome Staining

**Figure 3:**
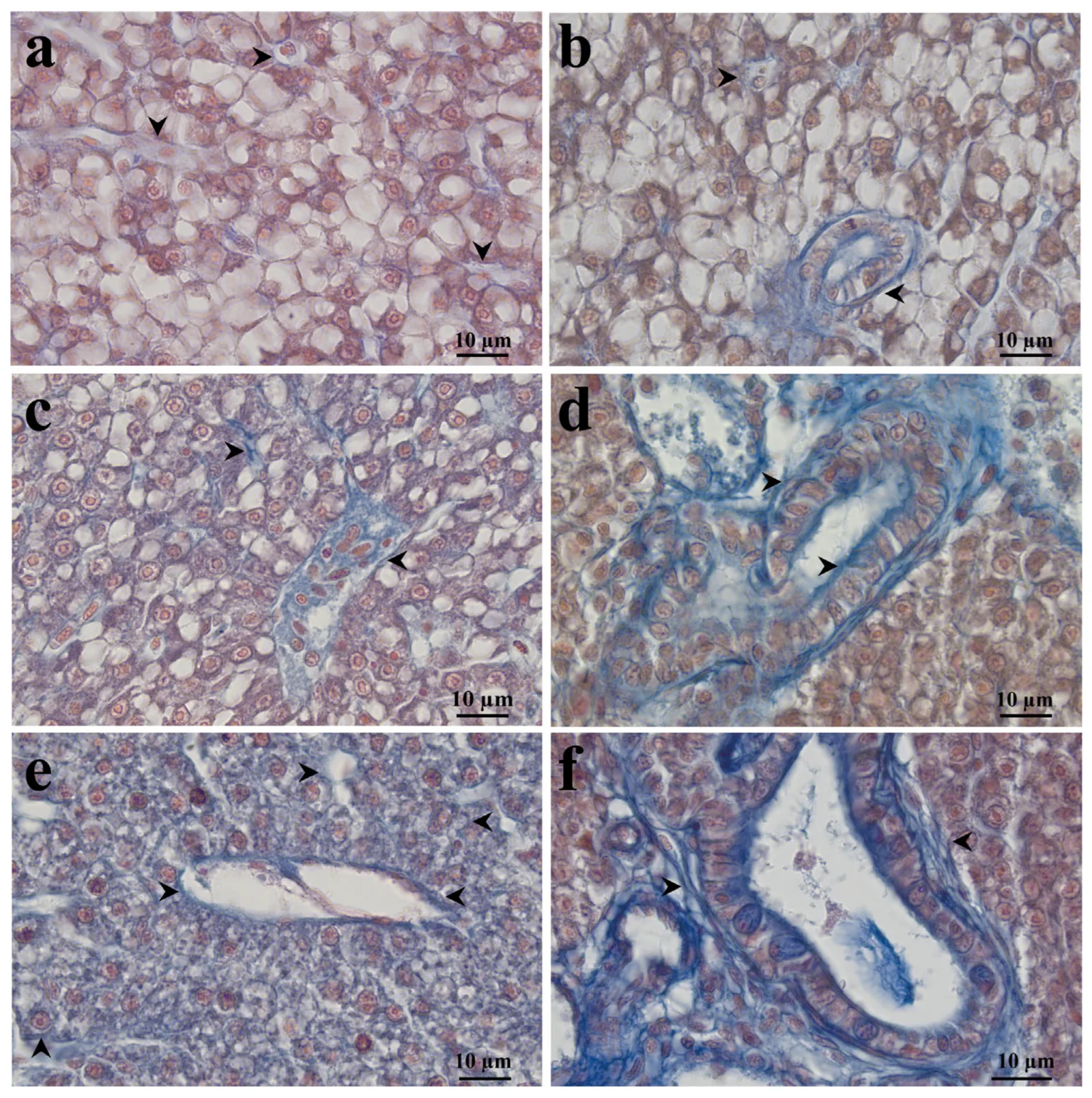
Light micrographs of *Danio rerio* liver (Azan Trichrome Staining). (**a**,**b**) In the control group, blue-stained collagen fibres were occasionally visible surrounding sinusoids, blood vessels and bile ducts (arrows). (**c**,**d**) After 96 h of exposure to 6.56 µg/L of dimoxystrobin, intense blue staining could be observed in the bile ducts and in the endothelium of blood vessels and sinusoids (arrows). (**e**,**f**) After 96 h of exposure to 13.13 µg/L of dimoxystrobin, collagen accumulation drastically increased in the bile ducts and blood vessels and was also evident throughout the parenchyma (arrows). The original microscopy images are provided in Supplementary Materials, File S1.

In the control group, blue-stained collagen fibres were observed as thin structures surrounding sinusoids, blood vessels and bile ducts (Figure 3a,b).

In fish exposed to a low concentration of dimoxystrobin, collagen deposition increased significantly compared to the control (*p* < 0.001, Table 1). Blue staining was evident in the endothelium of blood vessels and sinusoids (Figure 3c), in the basal and apical surfaces of the bile ducts and in the connective tissue surrounding them (Figure 3d).

At the high concentration, collagen accumulation increased both in intensity and spatial distribution (*p* < 0.001) compared to the control and low-concentration groups (Figure 3e,f; Table 1). Blue-stained fibres were particularly evident in the bile ducts and blood vessels and appeared scattered throughout the parenchyma (Figure 3e,f).

Overall, Azan staining demonstrated a dose-dependent increase in collagen deposition affecting both stromal and parenchymal compartments.

### 3.4. Alcian Blue pH 2.5 Staining

**Figure 4:**
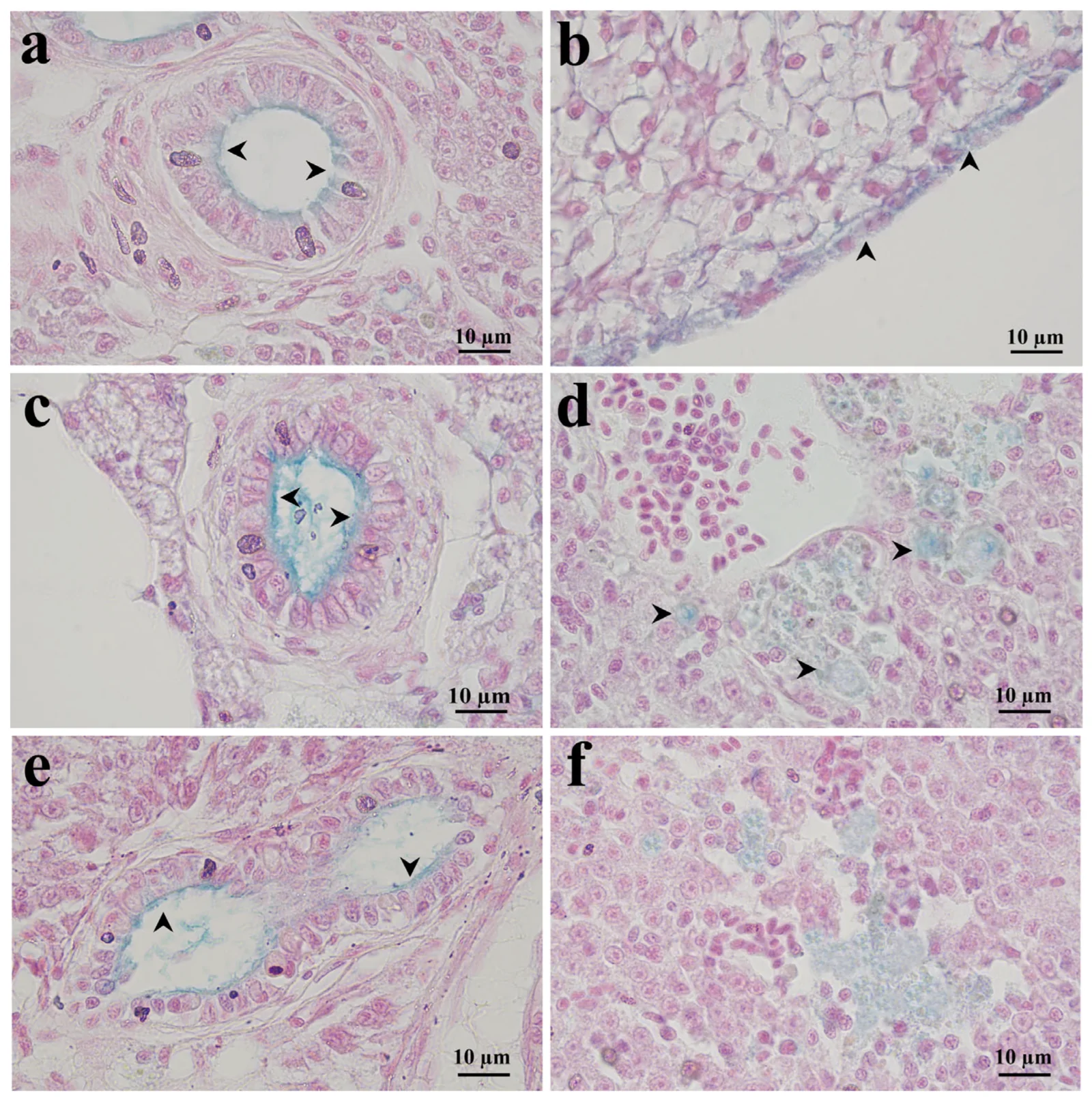
Light micrographs of *Danio rerio* liver (Alcian Blue pH 2.5 staining). (**a**,**b**) In the control group, a slight staining was visible only in the apical portion of the bile ducts (arrows). (**c**,**d**) After 96 h of exposure to 6.56 µg/L of dimoxystrobin, the staining increased, being visible in the apical portion of the bile ducts and in damaged areas of the parenchyma (arrows). (**e**,**f**) After 96 h of exposure to 13.13 µg/L of dimoxystrobin, the staining was comparable to that in the low-concentration group, as noted in both distribution pattern and intensity (arrows). The original microscopy images are provided in Supplementary Materials, File S1.

In the control group, a weak blue staining was observed in the apical portion of the bile ducts and scattered in the parenchyma, whereas sinusoids and blood vessels appeared unstained (Figure 4a,b).

In fish exposed to the low concentration of dimoxystrobin, blue staining increased significantly compared to the control (*p* < 0.001, Table 1). Alcian-positive deposits were observed in the bile ducts (Figure 4c) and near the structurally damaged area of the parenchyma (Figure 4d).

In the high-concentration group, the staining showed a significant increase compared with the control (*p* < 0.001, Table 1). The distribution pattern was similar to that observed in the low-concentration group and was particularly evident in the bile ducts (Figure 4e) and in some areas where the parenchyma exhibited disorganisation or structural loss (Figure 4f).

Overall, acidic glycosaminoglycan deposition increased following dimoxystrobin exposure, particularly around bile ducts and damaged hepatic regions.

### 3.5. PAS-A Staining

**Figure 5:**
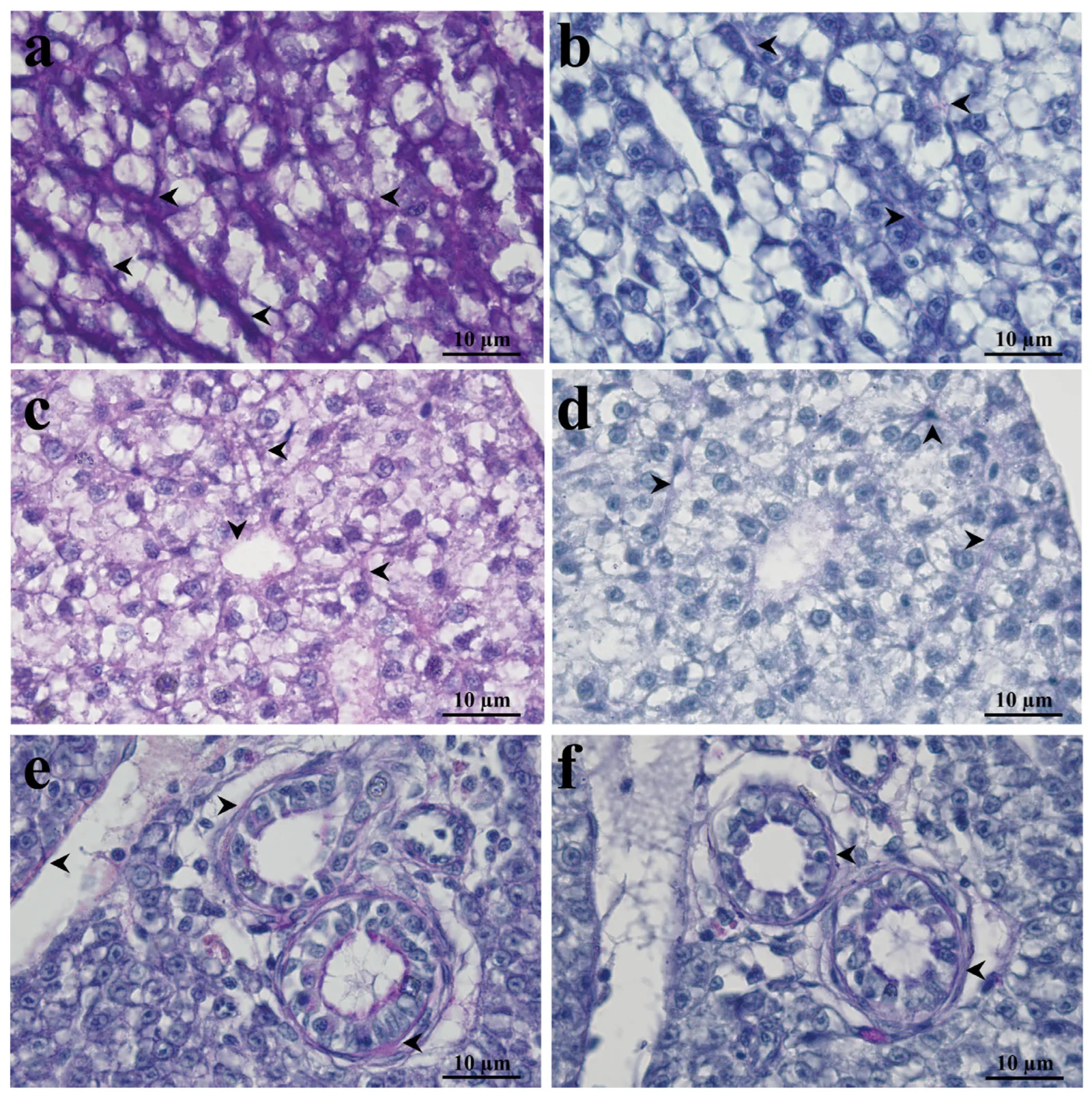
Light micrographs of *Danio rerio* liver (PAS-A staining). In the control group, intense magenta staining (arrows) was visible in the hepatocyte’s cytoplasm and in the endothelium of both vessels and sinusoids (**a**), while no staining was detected in amylase-treated sections (**b**). After 96 h of exposure to 6.56 µg/L of dimoxystrobin, a visible reduction in magenta staining (arrows) was noted (**c**); note in both parenchymal and stromal compartments a residual magenta staining (arrows) of amylase-resistant components (**d**). After 96 h of exposure to 13.13 µg/L of dimoxystrobin, PAS positivity further decreased, and a slight magenta staining (arrows) was detectable around blood vessels and bile ducts (**e**). Note the persistence of amylase-resistant PAS-reactive components (arrows) surrounding bile ducts (**f**). The original microscopy images are provided in Supplementary Materials, File S1.

PAS staining revealed intense and diffuse magenta positivity in the hepatocyte cytoplasm of the control group and in the endothelium of both vessels and sinusoids (Figure 5a; Table 2). The staining was almost completely abolished in the corresponding amylase-treated sections (Figure 5b), leaving only a minimal amylase-resistant PAS-positive area (Table 2).

In fish exposed to the low concentration of dimoxystrobin, non-amylase-treated sections showed a similar distribution of the staining (Figure 5c), although a visible reduction in the intensity of PAS positivity was noted compared with the control group (*p* < 0.001; Table 2). After amylase digestion, residual staining was observed in both parenchymal and stromal compartments (Figure 5d), consistent with semiquantitative analysis showing a significant increase in amylase-resistant PAS-positive area compared with controls (*p* < 0.001; Table 2).

In the high-concentration group, PAS positivity was markedly reduced in non-amylase-treated sections compared with both the control and low-concentration groups (*p* < 0.001, Table 2). Faint staining was observed around blood vessels and bile ducts and it was strongly reduced in hepatocytes (Figure 5e). Amylase-treated sections showed residual PAS positivity was mainly localised around the bile ducts (Figure 5f). Conversely, the amylase-resistant PAS-positive area was significantly increased compared with both the control and low-concentration groups (*p* < 0.001, Table 2).

Overall, PAS staining demonstrated progressive glycogen depletion accompanied by increased amylase-resistant PAS reactivity.

## 4. Discussion

The present study provides new evidence that short-term exposure to environmentally relevant concentrations of dimoxystrobin is associated with early alterations in hepatic tissue organization and extracellular matrix distribution in *Danio rerio*. A previous study from our research group demonstrated that dimoxystrobin affects the zebrafish liver, inducing morpho-functional alterations, oxidative stress, lipid accumulation and glycogen depletion [27]. In this context, the present study extends these findings by characterizing early extracellular matrix and hepatobiliary, with particular attention to collagen deposition, acidic glycosaminoglycans distribution, amylase-resistant PAS reactivity and bile pigment-like deposits. Together, these findings indicate that dimoxystorbin-induced hepatotoxicity involves coordinated alterations affecting both the hepatic parenchyma and the stromal microenvironment.

Hematoxylin and eosin staining confirmed that dimoxystrobin exposure compromised the general architecture of the zebrafish liver. The exposed groups showed progressive disorganisation of hepatic cords, hepatocyte swelling, cytoplasmic pallor, vascular congestion, bile duct epithelial alterations and, at the high concentration, mononuclear cell infiltration. These histopathological alterations are consistent with the primary mode of action of strobilurin fungicides. As QoIs, these compounds selectively block electron transport in mitochondria, inducing a severe bioenergetic collapse and a drastic reduction in ATP synthesis [45,46,47]. The resulting shortage of cellular energy impairs ATP-dependent ion pumps, triggering cellular degeneration, loss of cellular integrity, and cellular death [48,49,50,51]. Moreover, beyond hepatocellular degeneration, the structural alterations observed herein suggest that dimoxystrobin-induced liver injury also involves changes in the hepatic stromal microenvironment.

The structural hepatocellular alterations observed in the present study were accompanied by marked alterations in the hepatic extracellular matrix. Glycosaminoglycans are a structurally diverse class of linear polysaccharides that play essential roles in cell–matrix interactions, tissue organisation, and biological signalling, critically regulating cellular communication and tissue homeostasis [47,52,53]. Alterations in their distribution may reflect the pathological or physiological state of cells and tissues [54] and are considered among the earliest extracellular matrix responses to tissue injury and repair. In the present study, in the control group, the Alcian staining used to detect acidic glycosaminoglycan deposits was weakly restricted to the apical portion of the bile ducts. Following exposure to dimoxystrobin, glycosaminoglycan deposition increased markedly, extending into the connective tissue surrounding bile ducts and appearing as focal deposits adjacent to structurally damaged regions of the hepatic parenchyma. Glycosaminoglycans within the extracellular matrix act as key modulators of the immune response by binding and immobilising cytokines and chemokines on endothelial surfaces [55]. Altered glycosaminoglycan matrix establishes a functional microenvironment that directly promotes, targets, and supports leukocyte migration and activation during inflammatory processes [56]. Accordingly, mononuclear cell infiltration was particularly evident in fish exposed to the highest concentration of dimoxystrobin.

Another important finding of the present study was the increase in collagen deposition observed in both exposed groups. After dimoxystrobin exposure, collagen fibres were more evident around bile ducts and blood vessels, along sinusoids, and around hepatocytes, indicating an altered distribution of collagen within hepatic tissue. Liver fibrosis is generally defined as the excessive accumulation of extracellular matrix proteins, including collagen, resulting from liver injury [33,40,57]. Although liver fibrosis is typically considered a chronic endpoint [58], the present histochemical findings indicate that short-term dimoxystrobin exposure is associated with early perisinusoidal and periductal extracellular matrix remodelling. Moreover, since excessive collagen deposition is closely associated with inflammation and hepatocyte damage [59], the concurrent increase in collagen staining, parenchymal degeneration, and mononuclear cell infiltration is consistent with an early profibrotic tissue response induced by dimoxystrobin. Together, these findings suggest that extracellular matrix remodelling occurs in parallel with the metabolic disturbances observed following dimoxystrobin exposure. In fish, the liver is the principal site of glucose metabolism, regulating multiple pathways, including glycolysis, gluconeogenesis, glycogen synthesis, and glycogenolysis [60,61]. The intense and diffuse magenta staining observed throughout the parenchyma in the control group after PAS-A staining validated the abundance of healthy hepatic glycogen reserves. After dimoxystrobin exposure, the striking, dose-dependent depletion of glycogen stores provides direct histochemical evidence of a massive, substantial mobilisation of carbohydrate deposits through accelerated glycogenolysis [62]. Interestingly, while parenchymal glycogen is severely depleted in both treated groups, amylase staining revealed amylase-resistant PAS positivity. These findings may reflect a partial replacement of depleted glycogen stores by non-glycogen PAS-positive components, possibly as an adaptive or compensatory response to altered hepatic carbohydrate metabolism. These observations are consistent with our previous report of glycogen depletion in the liver of dimoxystrobin-exposed zebrafish [27] and further demonstrate that this metabolic disturbance is accompanied by changes in the composition of PAS-reactive components.

In addition to extracellular matrix and metabolic alterations, dimoxystrobin exposure also affected hepatobiliary-associated structures. Fouchet–Van Gieson staining showed a dose-related increase in bile pigment-like deposits involving hepatocytes and bile ducts in the low-concentration group, with deposits extending also into the sinusoids in the high-concentration group. Fouchet–Van Gieson staining is the most commonly used method for detecting bile pigments [63]. Bilirubin is the final product of heme destruction, and it has been recognised as a marker of liver injury [64]. It is largely derived from the breakdown of senescent or damaged erythrocytes and, to a lesser extent, from the breakdown of heme-containing proteins such as cytochromes P450, cytochrome b5, and catalase [65,66,67]. In fish, increased bilirubin accumulation typically reflects liver stress [68]. Under basal conditions, hepatocytes produce bile acids that are subsequently transported into the duodenum via the biliary tract [69], and the observation of pigment deposition may reflect altered hepatobiliary transport or impaired bile clearance [70]. Moreover, the presence of inflammatory cells may also contribute to biliary epithelial dysfunction and altered bile handling [71]. Given oxidative stress and mitochondrial dysfunction have been associated with altered hepatobiliary function following exposure to environmental contaminants, it is plausible that similar mechanisms contribute to the accumulation of bile pigment-like deposits observed following dimoxystrobin exposure. These mechanisms could contribute to impaired pigment handling and the accumulation of bile-like deposits in hepatic tissue. Although the precise mechanisms underlying this response remain to be elucidated, the present findings indicate that hepatobiliary alterations represent an additional component of dimoxystrobin-induced liver injury in adult zebrafish.

Collectively, the present findings indicate that dimoxystrobin-induced hepatotoxicity is not restricted to hepatocellular degeneration but involves a coordinated response affecting multiple hepatic compartments. The combined histological, histochemical, and semi-quantitative evidence demonstrates contaminant-induced alterations in the hepatic parenchyma, extracellular matrix, glycogen metabolism, and hepatobiliary structures, suggesting that these processes occur simultaneously during the early response to acute exposure. Rather than representing isolated pathological events, the observed collagen deposition, glycosaminoglycan redistribution, glycogen depletion and accumulation of bile pigment-like deposits appear to reflect interconnected components of an integrated tissue response to chemical injury.

These findings contribute to a more comprehensive understanding of pesticide-induced hepatotoxicity in fish and support the value of combining complementary histological, histochemical and semi-quantitative approaches to improve the detection and interpretation of early sublethal hepatotoxic effects in aquatic organisms. From an environmental perspective, the present findings are relevant because the tested concentrations fall within the range considered environmentally relevant for dimoxystrobin in surface waters. Taken together with our previous evidence of dimoxystrobin-induced alterations in the gills and liver of adult zebrafish, as well as developmental, behavioural, and mitochondrial alterations in embryos and larvae, these results indicate that short-term dimoxystrobin exposure may affect multiple biological targets. In particular, altered extracellular matrix organisation and collagen deposition may compromise hepatic tissue architecture and potentially interfere with nutrient storage, xenobiotic metabolism, and regenerative processes, thereby reducing the ability of exposed fish to maintain physiological homeostasis and respond to additional environmental stressors. Although population-level effects were not directly assessed, such alterations, if sustained or repeatedly induced, could impair individual condition and potentially contribute to adverse ecological consequences.

Although dimoxystrobin’s approval has not been renewed in the European Union, its use in other countries, such as Brazil, highlights the need for measures to limit its transfer to aquatic environments, including no-spray buffer zones and vegetated filter strips [13]. However, because these measures may not fully mitigate the risk under all exposure scenarios, additional preventive and monitoring actions should also be considered.

## 5. Concluding Remarks, Limitations, and Future Perspective

Overall, the present findings show that short-term exposure to environmentally relevant concentrations of dimoxystrobin is associated with a complex pattern of hepatic histological and histochemical alterations in adult zebrafish that extends beyond hepatocellular degeneration. The combined histological, histochemical, and semi-quantitative evidence indicates a coordinated disruption of parenchymal architecture, extracellular matrix organisation, glycogen storage, and hepatobiliary-associated compartments. In particular, the convergence of collagen deposition, accumulation of acidic glycosaminoglycans, amylase-resistant PAS reactivity, and bile pigment-like deposits in periductal and stromal regions suggests that the biliary-associated microenvironment may represent an early and sensitive target of dimoxystrobin-induced hepatotoxicity. These findings highlight the value of integrating histological, histochemical, and semi-quantitative approaches to detect early liver injury induced by pesticides.

One limitation of this study is that it does not provide molecular identification of the specific components underlying the observed staining patterns. Future studies should therefore include molecular and biochemical endpoints targeting extracellular matrix remodelling. These analyses would help clarify whether the histochemical alterations observed here represent a transient adaptive response to acute hepatic injury or the early onset of a more persistent fibrogenic and hepatobiliary dysfunction process.

An additional limitation of this study is that sex was not evaluated as an independent biological variable. Consequently, potential differences between males and females in the magnitude or pattern of dimoxystrobin-induced hepatic alterations cannot be excluded; thus, future studies evaluating sex-specific differences may be warranted.

Despite these limitations, the present study provides the first evidence that dimoxystrobin exposure induces early extracellular matrix and hepatobiliary histochemical alterations in adult zebrafish liver, broadening the current understanding of its hepatotoxicity beyond isolated hepatocellular injury toward a more integrated model of parenchymal–stromal–biliary remodelling.

## Figures and Tables

**Table 1:** Semi-quantitative assessment of histochemical staining in hepatic tissue of control and dimoxystrobin-exposed groups.

	Ctrl	Low Concentration	High Concentration
Fouchet–Van Gieson	1.11 ± 0.62	4.38 ± 0.24 ^(a^***^)^	6.32 ± 0.47 ^(ab^***^)^
Azan Trichrome	14.47 ± 0.47	26.06 ± 0.13 ^(a^***^)^	36.94 ± 0.25 ^(ab^***^)^
Alcian Blue pH 2.5	2.36 ± 0.14	4.74 ± 0.42 ^(a^***^)^	5.14 ± 0.36 ^(a^***^)^

The values indicate the percentage of the section’s area that expresses staining ± standard deviation. ^a^ = significant difference between treated group and control group; ^b^ = significant difference between high concentration and low concentration group; *** *p* < 0.001.

**Table 2:** Semi-quantitative assessment of PAS-reactive components and glycogen-dependent PAS positivity in hepatic tissue of control and dimoxystrobin-exposed groups.

	Ctrl	Low Concentration	High Concentration
Total PAS-positive area	61.83 ± 0.25	34.54 ± 0.50 ^(a^***^)^	28.69 ± 0.50 ^(ab^***^)^
Amylase-resistant PAS-positive area	3.91 ± 0.11	6.15 ± 0.15 ^(a^***^)^	12.64 ± 0.04 ^(ab^***^)^

^a^ = significant difference between treated group and control group; ^b^ = significant difference between high concentration and low concentration group; *** *p* < 0.001.

## Data Availability

The original contributions presented in this study are included in the article/Supplementary Materials. Further inquiries can be directed to the corresponding author.

## Supplementary Materials

The following supporting information can be downloaded at: https://www.mdpi.com/article/10.3390/jox16040148/s1, Table S1: Statistical comparison of replicate tanks for Fouchet–Van Gieson staining; Table S2: Statistical comparison of replicate tanks for Azan Trichrome staining; Table S3: Statistical comparison of replicate tanks for Alcian Blue pH 2.5 staining; Table S4: Statistical comparison of replicate tanks for Total PAS-positive area; Table S5: Statistical comparison of replicate tanks for amylase-resistant PAS-positive area; File S1: Original microscopy images.
