# Supplementary material for: Histochemical and Semi-Quantitative Evidence of Dimoxystrobin-Induced Liver Extracellular Matrix Remodelling in Adult Zebrafish
Source: J Xenobiot. 2026 Aug 11;16(4):148. doi: 10.3390/jox16040148 (PMC13514401; doi:10.3390/jox16040148)
Supplement: Supplementary file 1 [file jox-16-00148-s001.zip › jox-4438626-supplementary.pdf]

Histochemical and semi-quantitative evidence of dimoxystrobin-induced liver extracellular matrix remodelling in adult zebrafish

Ilaria Olivito, Valentina Basile, Antonio Paolo Maria Graziani, Naouel Gharbi and Rachele Macirella

Table S1. Statistical comparison of replicate tanks for Fouchet-Van Gieson staining.

Experimental Group	Tank ID	N	Mean ± SD	Exact p-value
**Control**	Tank 1	7	1.13 ± 0.63	0.7978
	Tank 2	7	1.17 ± 0.61	
	Tank 3	7	1.02 ± 0.70	
	**Pooled**	**21**	**1.11 ± 0.62**	
**Low concentration**	Tank 1	7	4.39 ± 0.25	0.8978
	Tank 2	7	4.36 ± 0.23	
	Tank 3	7	4.39 ± 0.24	
	**Pooled**	**21**	**4.38 ± 0.24**	
**High concentration**	Tank 1	**7**	6.30 ± 0.49	0.9375
	Tank 2	**7**	6.34 ± 0.45	
	Tank 3	**7**	6.32 ± 0.48	
	**Pooled**	**21**	**6.32 ± 0.47**	

Table S2. Statistical comparison of replicate tanks for Azan Trichrome staining.

Experimental Group	Tank ID	N	Mean ± SD	Exact p-value
**Control**	Tank 1	7	14.45 ± 0.48	0.8871
	Tank 2	7	14.49 ± 0.46	
	Tank 3	7	14.47 ± 0.49	
	**Pooled**	**21**	**14.47±0.47**	
**Low concentration**	Tank 1	7	26.05 ± 0.14	0.9118
	Tank 2	7	26.07 ± 0.12	
	Tank 3	7	26.06 ± 0.14	
	**Pooled**	**21**	**26.06±0.13**	
**High concentration**	Tank 1	**7**	36.92 ± 0.26	0.8503
	Tank 2	**7**	36.96 ± 0.24	
	Tank 3	**7**	36.94 ± 0.25	
	**Pooled**	**21**	**36.94±0.25**	

Table S3. Statistical comparison of replicate tanks for Alcian Blue pH 2.5 staining.

Experimental Group	Tank ID	N	Mean ± SD	Exact p-value
**Control**	Tank 1	7	2.37 ± 0.15	0.8480
	Tank 2	7	2.34 ± 0.13	
	Tank 3	7	2.37 ± 0.14	
	**Pooled**	**21**	**2.36±0.14**	

**Low concentration**	Tank 1	7	4.75 ± 0.43	0.8998
	Tank 2	7	4.71 ± 0.40	
	Tank 3	7	4.76 ± 0.44	
	**Pooled**	**21**	**4.74±0.42**	
**High concentration**	Tank 1	**7**	5.12 ± 0.38	0.8920
	Tank 2	**7**	5.17 ± 0.34	
	Tank 3	**7**	5.13 ± 0.37	
	**Pooled**	**21**	**5.14±0.36**	

Table S4. Statistical comparison of replicate tanks for Total PAS-positive area.

Experimental Group	Tank ID	N	Mean ± SD	Exact p-value
**Control**	Tank 1	7	61.81 ± 0.26	0.8503
	Tank 2	7	61.85 ± 0.24	
	Tank 3	7	61.83 ± 0.25	
	**Pooled**	**21**	**61.83±0.25**	
**Low concentration**	Tank 1	7	34.52 ± 0.52	0.9158
	Tank 2	7	34.57 ± 0.48	
	Tank 3	7	34.53 ± 0.51	
	**Pooled**	**21**	**34.54±0.50**	
**High concentration**	Tank 1	**7**	28.67 ± 0.51	0.8604
	Tank 2	**7**	28.71 ± 0.49	
	Tank 3	**7**	28.69 ± 0.51	
	**Pooled**	**21**	**28.69±0.50**	

Table S5. Statistical comparison of replicate tanks for Amylase-resistant PAS-positive area.

Experimental Group	Tank ID	N	Mean ± SD	Exact p-value
**Control**	Tank 1	7	3.90 ± 0.12	0.8478
	Tank 2	7	3.93 ± 0.10	
	Tank 3	7	3.90 ± 0.12	
	**Pooled**	**21**	**3.91±0.11**	
**Low concentration**	Tank 1	7	6.14 ± 0.16	0.8491
	Tank 2	7	6.17 ± 0.14	
	Tank 3	7	6.14 ± 0.16	
	**Pooled**	**21**	**6.15±0.15**	
**High concentration**	Tank 1	**7**	12.63 ± 0.05	0.7513
	Tank 2	**7**	12.65 ± 0.03	
	Tank 3	**7**	12.64 ± 0.04	
	**Pooled**	**21**	**12.64±0.04**	

In all Tables, data are presented as mean ± standard deviation. The Kruskal-Wallis test was used to evaluate differences among replicate tanks within the same experimental group.
